# Safety and Observations in a Pilot Study of Lenalidomide for Treatment in Autism

**DOI:** 10.1155/2012/291601

**Published:** 2012-09-11

**Authors:** Michael Chez, Renee Low, Carol Parise, Tammy Donnel

**Affiliations:** ^1^Sutter Neuroscience Medical Group, 1625 Stockton Boulevard, Suite 104, Sacramento, CA 95816, USA; ^2^Sutter Institute for Medical Research, 2801 Capitol Avenue, Suite 400, Sacramento, CA 95816, USA

## Abstract

Autism affects 1 : 88 children in the United States. Familial history of autoimmune disease, autoantibodies in the serum of mothers when there is more than one autistic offspring, and neuroglial response in CSF and brain tissue in autistic patients suggest an immunological variable may be associated with this condition. Lenalidomide has the potential to invoke changes in TNF-**α** with less toxicity than thalidomide. This pilot study evaluated lenalidomide at reduction of TNF-**α** and improvement of behavior and language in children with autism with elevated TNF-**α**. Subjects with elevated TNF-**α** were given 2.5 mgs lenalidomide daily for 12-weeks. Pharmacodynamics and safety was evaluated. Changes in language and autistic behaviors after six and twelve weeks were measured. Although statistical significance was not achieved for most measures, there were trends toward improvement. After 6-weeks, mean receptive language increased: 60.67 ± 12.06 to 65.00 ± 15.10 (*P* = 0.11) and symptoms of autism decreased (40.75 ± 5.96 versus 38.67 ± 7.90, *P* = 0.068). After 12-weeks, CSF-TNF-**α** declined 57% ± 25% from 80.5 ± 41.03 to 38.0 ± 31.27 (*P* = 0.068). Serum TNF-**α** declined 57% (92.50 ± 68.92 to 40.25 ± 44.53 (*P* = 0.048). This study suggests that lenalidomide is tolerated as a treatment by children with autism and should be further studied as a potential agent for cytockine inflammation.

## 1. Introduction

Autism is currently the leading cause of developmental disability in the United States and most other countries of the world. The condition currently affects 1 in 88 children born in the United States as of 2010 [1]. Autism is best defined as a spectrum of heterogeneous developmental disabilities mainly involving three core aspects of behaviors: (1) speech and communication; (2) social interest and interaction; (3) stereotypic behaviors or mannerisms [2]. Historically, the incidence of autism has increased; however, debate exists as to whether this reflects simple population growth, recategorization and increased recognition, or whether there is a true increase in the percent of the population affected [3]. Despite this controversy, most experts and the general population agree that the incidence of autism has greatly increased, especially in states such as California which has shown a massive increase of 600% for autistic children in state educational records over two decades [4–6].

There is currently no agreed upon single genetic or other etiological risk factor that has been shown to cause autism in isolation. Current thinking is that multiple risk factors such as familial, environmental such as extreme prematurity or infection, and immune triggers may need to occur.

Patients with autism may have a dysfunctional immune system, especially in the nervous system. There is evidence of serum markers for inflammation including elevation of cytokine, autoantibodies, lower levels of normal immunoglobulins for immune defense, and neuroglial and cytokine activation in cerebral spinal fluid (CSF) or brain tissue [7, 8]. Innate neuroglial immune dysregulation has been shown with elevation of interleukin-6 and other proinflammatory markers in the frontal cingulate cortex and CSF, as described by Vargas et al. and others [9–11]. Only one study looking at cytokine levels in CSF and serum in autism has been completed that describes an elevated TNF-*α* ratio of the cerebrospinal fluid levels to serum levels [7].

Lenalidomide, an analogue of thalidomide, has the potential to invoke more significant changes in TNF-*α* and other immunomodulatory cytokines with less toxicity than thalidomide. Lenalidomide may be clinically viable as an oral agent early in the course of autism subtypes and may be preferred over injectable agents. Lenalidomide may be useful in modifying the course of the disease in patients with elevated tumor necrosis factor-alpha (TNF-*α*) in serum or CSF as well as those patients with other elevated cytokine profile such as Interleukin 1 (IL-1), Interleukin 6 (IL-6), or methyl CpG-binding protein 1 (MeCP-1).

The purpose of this phase 2 pilot study is to determine the safety and pharmacodynamics of lenalidomide in children with autism who have elevated TNF-*α* levels. We also examine the potential of lenalidomide to reduce TNF-*α* and improve behavior and speech function.

## 2. Methods

### 2.1. Subjects

Subjects consisted of 7 males aged 6 to 12 years with a diagnosis of autism as defined by the DSM-IV-TR and a history of regression in their language and social abilities reported by their parents. The patients had a history of autoimmune dysfunction either in a first-degree relative or in their mother either during or after the pregnancy. Because of the history of regression, a thorough evaluation was done in all patients prior to this study to evaluate their autism and rule out degenerative disease. This evaluation included neuroimaging, 24-hour EEG, genetic testing, metabolic testing, and sampling of serum and CSF to obtain cytokine panels. Patients with elevated CSF TNF-*α* (>50 pg/mL) noted 0–24 months prior to study onset were asked to be screened for this study. Because a higher percent of males versus females are diagnosed with autism and the potential teratogenic effect of lenalidomide, only males were chosen for this study. Patients with the following conditions were excluded from the study: PPD-NOS and Aspergers by DSM-IV-TR, genetic disorders, active autoimmune conditions such as Crohn's or Hashimoto's thyroiditis, or any hematological, hepatic or renal condition that would place subjects at unacceptable risk if they were to participate.

Participation was voluntary and without compensation. A Food and Drug Administration Investigational New Drug application was approved for this study (IND102897). Written informed consent was obtained by the study coordinator from at least one parent of subjects at the baseline visit. The study was approved by the Sutter Health Central Institutional Review Committee. Patient pharmacodynamic data was collected and analyzed by the Celgene Laboratory. Cytokine measurements were analyzed by InterScience Laboratory, Inglewood, CA (CLIA approved), using their commercial protocol for serum and CSF which was frozen and shipped to them.

### 2.2. Drug and Dosing

Lenalidomide 2.5 mgs was given daily for 12 weeks. This low dose was selected to minimize the risk of adverse effects. In addition, because this was a pilot study, the goal was to test the lowest dose that could potentially lead to improvements.

### 2.3. Measurements

Baseline behavioral and neuropsychological evaluations included the Receptive and Expressive One-Word Picture Vocabulary Tests, Autism Diagnostic Observation Schedule (ADOS), the Childhood Autism Rating Scale (CARS), as well as a Clinical Global Impression Improvement (CGI-I). Behavioral and neuropsychological evaluations were measured at the baseline visit and again 6 weeks and 12 weeks after baseline.

#### 2.3.1. Receptive and Expressive One-Word Picture Vocabulary Test

The Receptive One-Word Picture Vocabulary Test [12] is an individually administered, norm-referenced test that provides an assessment of an individual's English hearing vocabulary. For each item, examinees are presented with a word, spoken by the examiner, along with four illustrations. Examinees select the illustration that depicts the meaning of the word. When examinees are unable to correctly identify the pictured meaning of the word in six out of eight consecutive items, testing is discontinued.

For the Expressive One-Word Picture Vocabulary Test [13] examinees are shown a series of illustrations and asked to name each as it is presented. When the examinee is unable to correctly name six consecutive items, testing is discontinued.

Both the Expressive and Receptive One-Word Tests are standardized for use with individuals 2 through 18 years of age.

#### 2.3.2. Autism Diagnostic Observation Schedule (ADOS)

The Autism Diagnostic Observation Schedule (ADOS) [14] is a semistructured, standardized assessment of communication, social interaction, and play or imaginative use of materials for individuals with possible autism or other pervasive developmental disorders. The ADOS consists of standard activities that allow the examiner to observe behaviors that have been identified as important to the diagnosis of autism spectrum disorders at different developmental levels and chronological ages. The ADOS consists of four modules and each module has its own protocol designed for use with children or adults at a particular developmental and language level, ranging from no expressive language to verbally fluent adults. The examiner selects the module that is most appropriate for a particular child or adult on the basis of his/her expressive language skills and chronological age. Overall ratings are completed immediately after administration. These ratings can then be used to formulate a diagnosis through the use of the diagnostic algorithm provided for each module.

#### 2.3.3. The Childhood Autism Rating Scale

The Childhood Autism Rating Scale (CARS) [15] is used to assess changes in symptoms of autistic disorder. This 15-item behavior rating scale helps to identify children with autism and to distinguish them from other diagnoses. Each item covers a particular characteristic, ability, or behavior. After observing the child and examining relevant information from parent reports and other records, the examiner rates the child on each item. Using a 7-point scale, he or she indicates the degree to which the child's behavior deviates from that of a normal child of the same age. After the child has been rated on each of the 15 items, a total score is computed by summing the individual ratings. Children who score above a given point are categorized as autistic. In addition, scores falling within the autistic range can be divided into two categories—mild-to-moderate autism and severe autism. Decreased scores on this rating scale reflect improvement in symptoms.

#### 2.3.4. Clinical Global Impression-Improvement

The investigator evaluated each child using the clinical global impression score (CGI-I) at baseline, six weeks, and twelve weeks. The CGI is designed to reflect changes in core autism symptoms and behaviors over multiple examinations [16]. Decreased scores on this rating scale reflect improvement in symptoms.

#### 2.3.5. TNF-*α* Assay

CSF and serum TNF-*α* were measured up to 21 months prior to baseline. Serum TNF-*α* was obtained again at the 12-week visit. CSF TNF-*α* was also measured in children whose parents opted for a lumbar puncture. The lumbar puncture was conducted after patients were admitted into the pediatric outpatient treatment unit of Sutter Memorial Hospital (Sacramento, CA) and the parent or guardian signed an additional consent granting permission to perform the procedure. An anesthesiologist monitored and sedated the child with propofol to allow a safe and painless procedure for obtaining CSF for cytokine values. A topical anesthetic was applied at the site of the puncture and the puncture site was cleaned with gauze pads soaked in antiseptic solution. After the anesthetic took effect the patient had the procedure performed and 5–10 mL of CSF was removed for analysis as above, and the child was monitored until safely released from the procedure per the attending anesthesiologist. The CSF cell count, protein, and glucose were obtained simultaneously so that no other inflammatory process was present. Patients where the CSF was obtained more than 12 months prior to baseline (*n* = 2) had the option to repeat the CSF levels. Both CSF levels were repeated for both patients. All patients completing the treatment trial had CSF levels and serum levels for TNF-*α* repeated per protocol (*n* = 4).

### 2.4. Specimen Collection and Preparation

The CSF and serum samples were frozen after collection and analyzed by Inter Science Institute (ISI), Inglewood, CA (CLIA certification 05D0549406) using a solid phase ELISA technique with a monoclonal antibody specific for TNF-*α* (R&D systems/Quantikine). Three in-house controls with high, medium, and low concentrations were included in the assay. As part of standard preanalytical procedures, any CSF samples that were observed to contain sediment were centrifuged at 1300 g for 5 minutes to remove any particulate matter. The assay was otherwise performed according to the standard protocol. Specimens were diluted 1 : 1 and 1 : 10 for five patients at baseline and four patients at 12 weeks, except for two patients who had only the lower diluted values, to establish comparative values for TNF-*α*. Serial dilution linearity had been previously validated up to 1 : 32. A standard point was added by an additional dilution to extend the lower range of reporting. The CSF and serum of patients were analyzed and ratios were calculated.

### 2.5. Analytical Sensitivity and Reproducibility of ISI's TNF-*α* Assay

Sensitivity of the ISI assay was established over 10 replicates. The mean sensitivity was 0.298 pg/mL with an LOD of 0.3133 pg/mL. Interassay and intra-assay variation were established using high, medium, and low control materials. In 20 consecutive assays, the target values for interassay variation of the control materials were 21.7, 323.5, and 628.2 pg/mL with coefficient of variation (CV) of 9.2, 3.5, and 1.7%, respectively. The intra-assay variation had target values of 21.8, 333.3 and 621.0 pg/mL, respectively, with CV's at 9.8, 2.7 and 1.5% respectively. The linearity of the assay had an *r* ^2^ value of 0.9999 with an *r* of 0.9999 and a slope of 0.9903; the recovery observed values had similar *r*-values with a slope of 0.9992 and average recovery of 102%.

### 2.6. Pharmacokinetic Testing

At the baseline visit, patients were checked into an outpatient pediatric center for the day and given a brief physical. Subject's parents were given the choice to have a saline lock for blood draws or to have four venipunctures for the pharmacokinetic samples to be drawn. The samples were collected at 1, 2, 4, and 8 hours after first study drug dose. Blood samples were collected in a 6 mL K2-EDTA tube. Samples were immediately immersed in ice. Within 15 minutes of collection time, sample was taken to the laboratory and placed in refrigerated centrifuge at 1500 g for 10 minutes at 4°C. Plasma was aliquoted into 2 prelabeled cryovials. Within 30 minutes of blood collection time, the cryovials were placed in −20°C freezer. The samples were kept frozen and sent for analysis in batches. The validated HPLC-MS/MS methods were used to determine lenalidomide concentrations in human plasma samples. Lenalidomide was analyzed after the liquid-liquid extraction (with methyl *tert*-butyl ether (MtBE): ethyl acetate, 50 : 50, v/v) from 0.2 mL of human plasma samples. The reference standard of lenalidomide and stable isotope-labeled internal standard [^13^C_5_]-CC-5013 were synthesized by Celgene Corporation (Summit, NJ). HPLC was performed on a Synergy Polar-RP 4-*μ*m column (Phenomenex, Torrance, CA, USA). A triple quadrupole mass spectrometer (API 4000, Applied Biosystems, Foster City, CA, USA) was used for mass analysis and detection. Quantification was performed using multiple-reaction monitoring (*m*/*z* of 260.3/149.2 for lenalidomide and 265.1/149.2 for the IS) with electrospray ionization in the positive ion mode. The lenalidomide standard curve was linear (*r* ^2^ > 0.99) from 1 to 200 ng/mL in plasma. The lower limit of quantitation was 5 ng/mL in plasma. Validation quality control samples at 4 concentrations (1, 3, 60, and 180 ng/m) were used to determine assay accuracy and precision. Interday assay accuracy ranged from −1.3 to 6.5% deviation from the nominal concentration. Interday precision did not exceed 8.0% CV in the method validation. All samples were analyzed by QPS (Newark, DE, USA).

### 2.7. Treatment

Lenalidomide 2.5 mgs was given daily for 12 weeks. Subjects had a weekly study visit after administration of the first dose. A parent was given instructions on how to administer lenalidomide including precautions for medication handling. Subsequent doses were administered daily by the parents of the subjects. At the beginning of the visit, laboratory testing was conducted and processed. The investigator performed a short physical and documented a CGI-I. Upon completion of a physical exam and receipt of laboratory results to assess conditions that may be related to drug toxicity, medication was dispensed for the next week.

### 2.8. Statistical Analysis

Data were graphically displayed and descriptive statistics used to assess the distribution of the measurements. The Wilcoxon signed-ranks test was used to analyze differences in the measurements between baseline, 6, and 12-week followup. The area under the curve (AUC), maximum observed concentration (*C*_max⁡_) and sampling time (hour) at which *C*_max⁡_ occurred (*t*_max⁡_) were used to evaluate the results of the pharmacokinetic testing.

## 3. Results

Seven white males aged 6–12 were enrolled in the study (Table 1). All children had parental history reporting regression between 12 and 18 months with loss of eye contact, verbal ability, and loss of nonverbal gesturing in 4/7 patients. No children regressed during the course of the study. Baseline CSF TNF-*α* in one of the seven children was processed using 1 : 1 dilution, the rest with 1 : 10 dilutions based on laboratory recommendations (InterScience Laboratory). Three children had initial observed rote and simple 1-2 word phrased speech, mostly with prompting, while four children were nonverbal. These lower functioning children were most likely why outcomes were harder to observe for language change. Prestudy serum and CSF TNF-*α* values were obtained 8.5 ± 5.7 months prior to baseline repeat serum levels. One child's initial sample was obtained 20.7 months prior to the start of the study but the repeat results were obtained within 9 months of the baseline visit. Serum TNF-*α* was processed with 1 : 1 dilution for two children and 1 : 10 dilution for five children. All patients had serum levels of cytokines done at baseline and one patient had CSF sample repeated.

**Figure 1:**
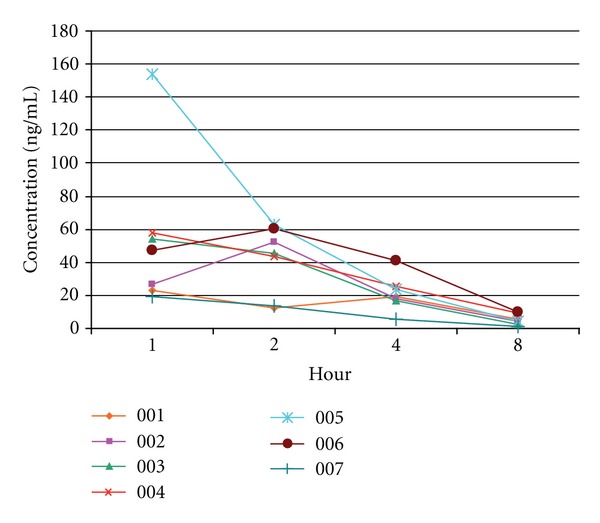
Concentration of 2.5 mgs lenalidomide up to eight hours after administration of first dose.

Pharmacokinetic (PK) data were obtained for 7 subjects (Table 2 and Figure 1). Six of the seven children had serum concentrations between 20 and 60 pg/mL one hour after the first dose. Eight hours after the first dose, the serum concentration for all children was less than 20 pg/mL. The PK value for subject 5 was significantly higher than all other subjects at the 1-hour mark but this normalized at 2 hours. Table 2 provides the parameter estimates for each patient. Concentration peaked in the second hour for 2 subjects and in the first hour for the remaining 5.

Two patients developed a rash and were discontinued from the study. One patient was discontinued from the study after eight weeks for transient drop of absolute neutrophil count of 1200 (safety cutoff 1500). All rashes cleared after stopping lenalidomide, and ANC returned to normal levels after stopping in patient affected.

### 3.1. TNF-*α*

At the 12-week followup, 1 : 10 diluted serum TNF-*α* was obtained in three of the same children who had 1 : 10 diluted serum at baseline. One case processed using 1 : 1 dilution at baseline had his serum processed using 1 : 1 at the 12-week followup. One child's serum was processed using 1 : 1 dilution at baseline and was processed using 1 : 10 dilution at follow-up; therefore, this case was excluded from the analysis (protocol change from InterScience laboratory). CSF TNF-*α* values were consistently measured for four subjects who completed the study (Table 1). CSF-TNF-*α* declined an average of 57% ± 25% from 80.5 ± 41.03 to 38.0 ± 31.27 in four children whose samples were consistently measured and completed the study at 12 weeks (*P* = 0.068). Serum TNF-*α* showed decline of 57% (92.50 ± 68.92 to 40.25 ± 44.53; *P* = 0.068). Only TNF-*α* was used as an exploratory outcome measure because other cytokines were not always available in all patients. The investigator has also observed and reported of elevated CSF/serum ratios in autistic patients [7].

### 3.2. Language and Behavioral Measures

At the 6-week followup, mean receptive language in three of the six patients with initial phrase speech increased from 60.67 ± 12.06 to 65.00 ± 15.10 (*P* = 0.110) (Table 3). Six children who completed the 6-week followup showed decreased symptoms of autism based on the CARS scores that was significant for change (baseline 40.75 ± 5.96 versus 6 weeks 38.67 ± 7.90, *P* = 0.043).

Clinical Global Impression ratings by the investigator indicated some improvements in socialization, language expression, and receptive speech (Table 4). Mean baseline CGI expressive language was 5.43 ± 1.27 and was statistically improved at 6 weeks (4.00 ± 2.12, *P* = 0.042) and at 12 weeks (4.17 ± 1.60, *P* = 0.023). CGI assessment of receptive language showed a similar improvement between baseline (4.50 ± 1.56) and 6 weeks (2.50 ± 1.18, *P* = 0.027) but the improvements did not remain statistically significant at 12 weeks (3.33 ± 1.63, *P* = 0.066). While social skills scores improved at both 6 weeks (2.50 ± 1.00) and 12 weeks (3.00 ± 1.41), the differences were not significant compared to baseline (4.83 ± 1.17) *P* = 0.066.

## 4. Discussion

This was a pilot study to determine the safety and potential efficacy of lenalidomide in a small number of patients and was not intended to have adequate power to achieve statistically significant findings. The fact that the majority of the patients with history of regression showed almost no expressive ability at baseline is not atypical for some regressive subtypes seen clinically, but it limited the ability of the study to measure any significant change. This study suggests that the oral TNF-*α* inhibiting agent lenalidomide is a potential novel mechanism for treatment of autism with regression in the presence of elevated TNF-*α* markers in CSF and serum. Clear clinical lowering of TNF-*α* in both serum and CSF was observed in four patients completing the 12-week study. These findings build upon prior animal models of inflammatory cytokine elevation and change in rat offspring behavior with cytokine treatment [17, 18] and appear to have potential application in human patients based on these pilot study results. In addition to the number of subjects who completed the study, a limitation of this study included logistical limitations in capturing CSF data at exact baseline and followup, which caused variability in the timeline between these measurements. Due to this we cannot conclude that all the observed changes in CSF levels were due to therapy alone.

Pharmacokinetic data was successfully obtained and safety was monitored successfully during the trial. Variability was seen in one patient only in the pharmacokinetic data. Rashes that subsequently recovered were seen on 2 patients. In clinical practice with lenalidomide, rashes are a potential reflection of treatment effectiveness and many will continue treatment through a rash but this protocol specified discontinuation of treatment if a rash occurred. Mild absolute neutrophil count (ANC) decline to <1500 was the cutoff for the FDA safety requirements on this protocol. No hepatic, renal, hematologic or thrombotic changes other than this single ANC event occurred.

The results showed a trend towards improvement in language. Three of the seven patients with initial phrase speech were observed by parents and the investigator to show improvement in receptive language and CGI, reflecting significant levels of change in social and receptive language. These observations were validated through improvements in one-word receptive language testing scores, which showed clinically meaningful changes by 6 weeks of treatment that were measurable yet not statistically significant. There were an insufficient number of cases after 12 weeks of followup to compute statistics for expressive and receptive language scores. CARS improved on average greater than 2 points and showed significance after 6 weeks. These exploratory outcomes suggest some relationship to treatment were seen, but the small sample size in an open-label study limits conclusions of treatment affect.

In addition to the small sample size, this study has several limitations, one of which was the inconsistency with the laboratory processing of TNF-*α*, as well as the inconsistent timing between CSF samples. There is no consensus on standard laboratory tests that should be analyzed for children with autism. TNF-*α* and other cytokine measurements are done infrequently so there is no standardization in how these labs are processed. The current lack of commercially standardized TNF values and test protocols somewhat limits exact correlation; however CSF and serum TNF samples were processed in the same manner at baseline and 12 weeks in all patients, so we felt the values were valid for analysis in this study—especially serum levels which reflected true baseline versus end of treatment changes. In addition, serum level values could be correlated to behavioral and language scoring. Consistent testing standards are necessary and timing of samples before true meaningful cytokine data in autism can be assessed in treatment protocols.

Patients' repeated exposure to the tests and developmental maturation are potential limitations to the research findings. Further, some patients completed neuropsychological testing after lab work, the medical appointment with the physician, and additional wait time, which led to behavior noncompliance that likely affected test results. No patients suffered severe adverse reactions and all three patients who dropped out of study recovered fully from ANC levels and rashes.

Despite the limitations, to our knowledge, this open-label study represents the first attempt to treat autism by specifically targeting elevated innate inflammatory cytokine levels. Safety monitoring and pharmacokinetic data were successfully completed during this pilot study and exploratory observations of clinical and cytokine changes suggest a trend towards improvement. Correlating treatment outcomes with cytokine level changes may be a target in future autism spectrum treatment, especially in those with known maternal or postnatal immunological risk factors. Larger blinded and placebo-controlled studies assessing cytokine measurement and cytokine-targeted treatment in autism patients with TNF-*α* or other inflammatory cytokine elevation are warranted.

## Figures and Tables

**Table 1:** Demographic and TNF-alpha (pg/ml) data for study subjects.

ID	Age	Completed	Baseline TNF-alpha (pg/ml)		Week 12 TNF-alpha (pg/ml)	
			CSF	Serum	CSF	Serum
1	7	Y	103	74	24	20
2	12	Y	19^∗^	14^∗^	5^∗^	8^∗^
3	7	Y	101	15^∗^	78	54
4	11	Y	99	102	45	27
5	8	N	157	180		106
6	6	N	232	165		
7	8	N	55	58		

*P* value^∗†^					0.068	0.068

^∗^ 1: 1 diluted samples.

^†^ *P* values represent the results of the differences between baseline values and 6 and 12-month values using the Wilcoxon Signed Ranks Test.

**Table 2:** Parameter estimates for each subject who underwent pharmacokinetic testing at 1, 2, 4, and 8 hours.

ID	*C*_max⁡_ (ng/mL)	*t*_max⁡_ (h)	AUC_T_ (ng/mL)
1	23.35	1	112.35
2	52.06	2	167.36
3	54.41	1	179.19
4	57.74	1	219.72
5	153.57	1	327.54
6	60.64	2	281.29
7	19.46	1	59.39

Mean	60.18	1.29	192.41
SD	44.43	0.49	92.93

**Table 3:** Language and autism behavior ratings over the course of the study for each study participant.

ID	Expressive Language			Receptive Language			Childhood Autism Rating Scale^∗^		
	Baseline	Week 6	Week 12	Baseline	Week 6	Week 12	Baseline	Week 6	Week 12
1	0.0	0.0	0.0	9.0	10.0	5.0	51.0	51.0	51.0
2	1.0	1.0	2.0	14.0	13.0	20.0	44.0	43.0	43.0
3	85.0	67.0	76.0	72.0	79.0	60.0	33.0	30.0	27.0
4	38.0	36.0	38.0	27.0	36.0	46.0	42.0	41.5	39.0
5	35.0	46.0		48.0	49.0		35.0	33.0	
6	0.0			0.0			39.5		
7	59.0	65.0		62.0	67.0		39.5	33.5	

*P* value^∗†^		1.00	na^††^		0.110	na^††^		0.043	0.109

^∗^ Decreasing scores reflect increased improvement.

^†^ *P* values represent the results of the differences between baseline values and 6 and 12-month values using the Wilcoxon Signed Ranks Test.

^††^ Insufficient data to compute.

**Table 4:** Individual clinical global impression ratings over the 12-week study period.

ID	Language Expressive^∗^			Receptive Language^∗^			Social Skills^∗^		
	Baseline	Week 6	Week 12	Baseline	Week 6	Week 12	Baseline	Week 6	Week 12
1	7.0	6.0	6.0	6.0	4.0	6.0	7.0		4.0
2	7.0	6.5	6.0	5.5	4.0	4.0		4.0	4.0
3	4.0	1.5	2.0	2.0	1.5	2.0	4.0	2.0	1.0
4	5.0	5.0	4.0	4.0	2.0	2.0	4.0	3.0	2.0
5	4.0	3.0	3.0	3.0	2.0	2.0	4.0	2.0	
6	6.0		4.0	6.0		4.0	5.0		4.0
7	5.0	2.0		5.0	1.5		5.0	1.5	

^†^ *P* value^∗^		0.042	0.023		0.027	0.066		0.066	0.066

^∗^Decreasing scores reflect increased improvement.

^†^ *P*  values represent the results of the differences between baseline values and 6 and 12-month values using the Wilcoxon Signed Ranks Test.

## References

[1] Baio J (2012). Prevalence of autism spectrum disorders-autism and developmental disabilities monitoring network, 14 sites, United States, 2008. *MMWR Surveillance Summaries*.

[2] American Psychological Association (1994). *Diagnostic and Statistical Manual of Mental Disorders*.

[3] Shattuck PT (2006). The contribution of diagnostic substitution to the growing administrative prevalence of autism in US special education. *Pediatrics*.

[4] Cavagnaro A *Autistic Spectrum Disorders: Changes in the California Caseload An Update: June 1987–June 2007*.

[5] Croen LA, Grether JK, Hoogstrate J, Selvin S (2002). The changing prevalence of autism in california. *Journal of Autism and Developmental Disorders*.

[6] M.I.N.D. Institute (2002). *Report to the Legislature on the Principle Findings from the Epidemiology of autism in California*.

[7] Chez MG, Dowling T, Patel PB, Khanna P, Kominsky M (2007). Elevation of tumor necrosis factor-alpha in cerebrospinal fluid of autistic children. *Pediatric Neurology*.

[8] Connolly AM, Chez M, Streif EM (2006). Brain-derived neurotrophic factor and autoantibodies to neural antigens in sera of children with autistic spectrum disorders, Landau-Kleffner syndrome, and epilepsy. *Biological Psychiatry*.

[9] Vargas DL, Nascimbene C, Krishnan C, Zimmerman AW, Pardo CA (2005). Neuroglial activation and neuroinflammation in the brain of patients with autism. *Annals of Neurology*.

[10] Pardo CA, Vargas DL, Zimmerman AW (2005). Immunity, neuroglia and neuroinflammation in autism. *International Review of Psychiatry*.

[11] Zimmerman AW, Connors SL, Matteson KJ (2007). Maternal antibrain antibodies in autism. *Brain, Behavior, and Immunity*.

[12] Brownell R (2000). *Receptive One-Word Picture Vocabulary Test (ROWPVT)*.

[13] Brownell R (2000). *Expressive One-Word Picture Vocabulary Test (EOWPVT)*.

[14] Lord C, Rutter R, DiLavore PC, Risi S (2002). *Autism Diagnostic Observation Schedule (ADOS)*.

[15] Schopler E, Reichler RL, Renner BR (1988). *Childhood Autism Rating Scale (CARS)*.

[16] Guy W (1976). *Assessment Manual for Psychopharmacology*.

[17] Patterson P (2006). Pregnancy, immunity, schizophrenia, and autism. *Engineering Science*.

[18] Patterson PH (2005). *Modeling Features of Autism in Animals*.

